# Importance of Getting Enough Sleep and Daily Activity Data to Assess Variability: Longitudinal Observational Study

**DOI:** 10.2196/31807

**Published:** 2022-02-22

**Authors:** María Óskarsdóttir, Anna Sigridur Islind, Elias August, Erna Sif Arnardóttir, François Patou, Anja M Maier

**Affiliations:** 1 Department of Computer Science Reykjavík University Reykjavík Iceland; 2 Reykjavík University Sleep Institute School of Technology Reykjavík University Reykjavík Iceland; 3 Department of Engineering Reykjavík University Reykjavík Iceland; 4 Internal Medicine Services Landspitali University Hospital Reykjavík Iceland; 5 Department of Technology, Management and Economics DTU-Technical University of Denmark Copenhagen Denmark; 6 Oticon Medical Copenhagen Denmark; 7 Department of Design, Manufacturing and Engineering Management Faculty of Engineering University of Strathclyde Glasgow United Kingdom

**Keywords:** wearable technology, nearable technology, internet of health care things, sleep, Withings, study duration, establishing standards, seasonality, mHealth, digital health

## Abstract

**Background:**

The gold standard measurement for recording sleep is polysomnography performed in a hospital environment for 1 night. This requires individuals to sleep with a device and several sensors attached to their face, scalp, and body, which is both cumbersome and expensive. Self-trackers, such as wearable sensors (eg, smartwatch) and nearable sensors (eg, sleep mattress), can measure a broad range of physiological parameters related to free-living sleep conditions; however, the optimal duration of such a self-tracker measurement is not known. For such free-living sleep studies with actigraphy, 3 to 14 days of data collection are typically used.

**Objective:**

The primary goal of this study is to investigate if 3 to 14 days of sleep data collection is sufficient while using self-trackers. The secondary goal is to investigate whether there is a relationship among sleep quality, physical activity, and heart rate. Specifically, we study whether individuals who exhibit similar activity can be clustered together and to what extent the sleep patterns of individuals in relation to seasonality vary.

**Methods:**

Data on sleep, physical activity, and heart rate were collected over 6 months from 54 individuals aged 52 to 86 years. The Withings Aura sleep mattress (nearable; Withings Inc) and Withings Steel HR smartwatch (wearable; Withings Inc) were used. At the individual level, we investigated the consistency of various physical activities and sleep metrics over different time spans to illustrate how sensor data from self-trackers can be used to illuminate trends. We used exploratory data analysis and unsupervised machine learning at both the cohort and individual levels.

**Results:**

Significant variability in standard metrics of sleep quality was found between different periods throughout the study. We showed specifically that to obtain more robust individual assessments of sleep and physical activity patterns through self-trackers, an evaluation period of >3 to 14 days is necessary. In addition, we found seasonal patterns in sleep data related to the changing of the clock for daylight saving time.

**Conclusions:**

We demonstrate that >2 months’ worth of self-tracking data are needed to provide a representative summary of daily activity and sleep patterns. By doing so, we challenge the current standard of 3 to 14 days for sleep quality assessment and call for the rethinking of standards when collecting data for research purposes. Seasonal patterns and daylight saving time clock change are also important aspects that need to be taken into consideration when choosing a period for collecting data and designing studies on sleep. Furthermore, we suggest using self-trackers (wearable and nearable ones) to support longer-term evaluations of sleep and physical activity for research purposes and, possibly, clinical purposes in the future.

## Introduction

### Background

Sleep disorders and short sleep durations are some of the main health challenges in current times. Obstructive sleep apnea is one such disorder and is estimated to affect 1 billion adults worldwide [1]. Insomnia, defined as difficulties in initiating or maintaining sleep, outlines another common sleep disorder [2,3]. Short sleep duration, although not a sleep disorder, is also a major risk factor for adverse health effects and death [3-5]. The gold standard measurement setting for clinical assessment of sleep quality and sleep disturbances is the use of polysomnography for 1 night, typically performed in a hospital environment [6]. Polysomnography is expensive and requires individuals to sleep with several sensors attached to their face, scalp, and body, which is cumbersome [7,8]. Furthermore, data from such a study gives no indication on important routine aspects of sleep quality such as the average total sleep time (TST) of individuals, when they normally go to bed and wake up, whether they are affected by seasonal changes, or whether they have insomnia [9]. Instead, to assess free-living sleep conditions, multiple night recordings in the home environment need to be performed [9]. In the medical field, this is typically accomplished by using wrist actigraphy, which involves a small watch-like device with an embedded accelerometer that often also records ambient light conditions and skin temperature [9]. The use of actigraphy is accompanied by a subjective sleep log or a sleep diary. Clinical guidelines recommend that the individual wears the actigraphy for 7 to 14 days; however, 72 hours of recording is generally sufficient to bill for testing in the United States [9]. For research purposes, 5 to 7 days of actigraphy measurements are often used to assess sleep behavior [10]. These data are used to assess, for example, average sleep duration, chronotype (morningness vs eveningness, commonly referred to as *A-type* vs *B-type*), and similar sleep parameters of interest. This type of data can also be used to facilitate the analysis of individual sleep patterns and for clustering purposes to show trends at the group level [11].

### Consumer-Grade Self-Tracking Technologies

More recently, consumer-grade self-tracking technologies that facilitate sleep data collection over longer periods have emerged [12]. Wearable technology (wearables) is an umbrella term for body-worn connected sensors [8]. Smartwatches are an example of such wearables and can capture information similar to actigraphy. Often, they collect even a wider range of physiological signals, such as heart rate, skin temperature, and oxygen saturation [13-15]. Other self-tracking technologies are nearable technologies (nearables), which can also be used to monitor physiological signals by close approximation to the body. These are increasingly used in conjunction with wearables in health-related research studies [14-16]. For instance, and relevant to our study, they include connected mattresses to monitor sleep patterns in more detail [17]. In most cases, consumer-grade self-trackers are designed for the general purpose of activity tracking. However, their ability to monitor a broad range of physiological parameters means that they are now seriously being considered as alternatives to medical-grade technology for the monitoring of various clinical conditions [18,19]. In addition, the portability and affordability of these trackers open up opportunities for pursuing clinical research on larger cohorts of participants and for rethinking the implementation of remote monitoring care models in specific patient populations [20].

Recent years have seen a surge of research on sleep with consumer-grade self-trackers. Most of these studies focus on relating measurement from the wearable device to either mental or physical health and sometimes both [21,22]. In a few cases, the duration of data collection varies from days and weeks [23-26] to months and years [27,28]. In addition, large sample sizes obtained from the vast number of people who wear self-trackers in the general population have been leveraged to study and compare sleep patterns by age, gender, and BMI worldwide, as in the work presented by Jonasdottir et al [12]. In terms of duration of data collection, a similar study associated shorter sleep duration and greater variability of sleep duration with increased BMI [28]. Furthermore, the large amount of data collected with self-trackers has encouraged the use of advanced machine learning techniques and deep learning to predict clinical outcomes more robustly [29,31]. Although some studies have taken on the task of observing participants over a longer time span than the gold standard for clinical assessment of sleep quality and sleep disturbances, to our knowledge, only a single study has compared data collected over 1 week with data collected over 2 weeks, concluding that the shorter period is sufficient [24]. Although the sleep research community acknowledges the need for longer periods of data collection with wearable and nearable (nonwearable that is placed near the body) self-trackers, the question of whether the participants should wear self-trackers for a longer time than the gold standard to generate a more insightful portrait of their sleep patterns remains unanswered [11,31].

### Aims and Overview

The primary goal of this paper is to investigate whether the time span of 3 to 14 days is sufficient for data collection when performing sleep measurements at home using wearable and nearable sensors. We address the primary goal through the following research question: is 3 to 14 days of data collection sufficient to capture the sleep habits and fluctuations in sleep patterns of an individual in a reliable way for research purposes? Our secondary goals are to investigate whether there is a relationship between sleep quality, physical activity, and heart rate and whether individuals who exhibit similar activity and sleep patterns in general and in relation to seasonality can be clustered together. We address the secondary goals through the following three research questions:

1. Is there a relationship between sleep quality, physical activity, and heart rate?
2. Can individuals who exhibit similar activity be clustered together in an insightful manner?
3. Are there significant differences between sleep patterns of individuals that are affected by seasonality and daylight saving time (DST) clock changes?

Our a priori hypothesis is that 3 to 14 days’ worth of data are neither sufficient to capture a person’s sleep habits nor sufficient to observe fluctuations in sleep patterns that might be important for research purposes.

## Methods

### Data Collection

**Figure 1:**
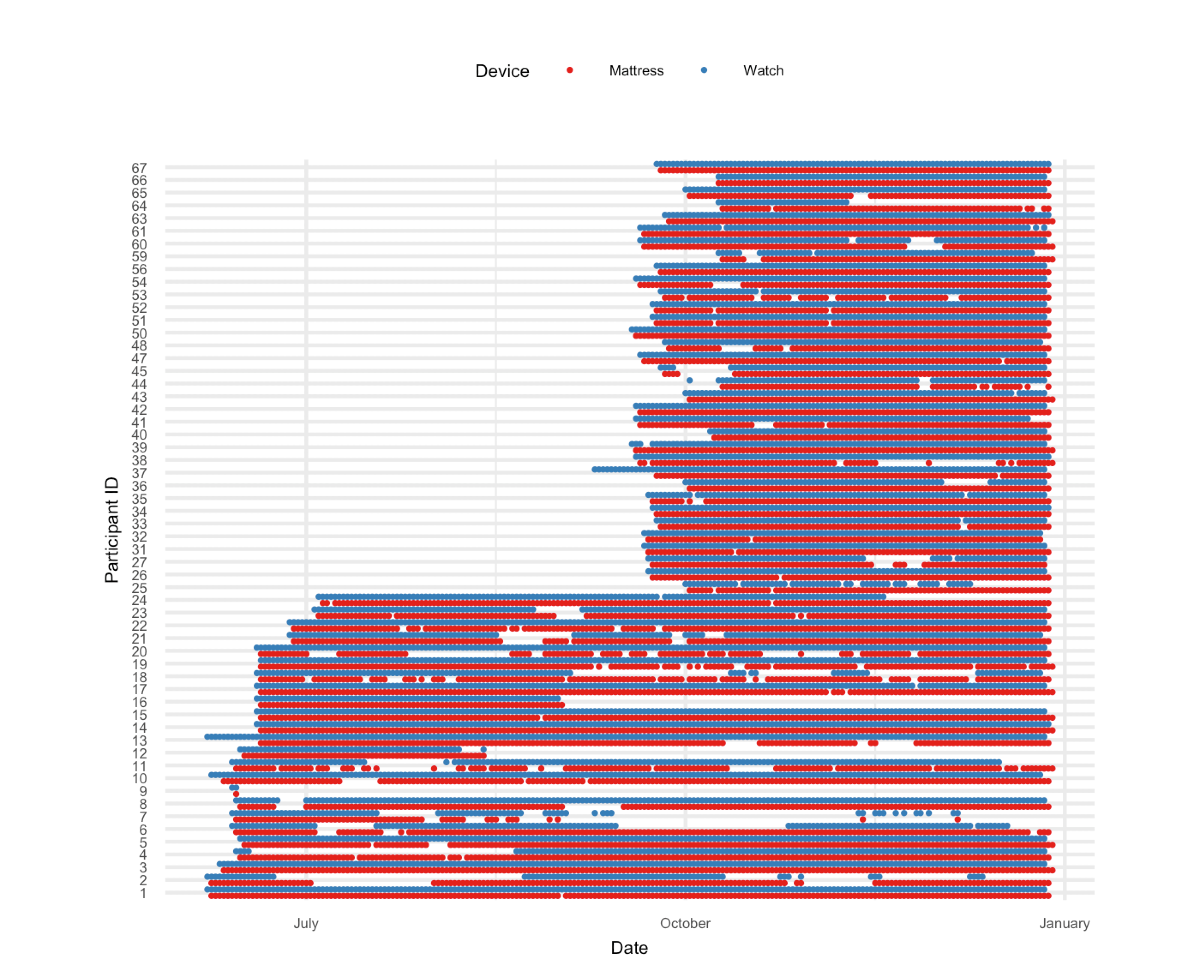
Overview of data collection for the participants in the study. The dots indicate dates with measurements. Blue represents periods with complete data from the smartwatch during the day, and red represents data from the sleep mattress during the night.

This study was proposed in the context of the Stanford Medicine X–Digital Health Challenge [32]. It was executed under an ethical waiver from the central Danish National Committee on Health Research Ethics. The participants were recruited through advertisements in 2 local newspapers (*Søndagsavisen Vestegnen* and *Villabyerne*) distributed within Greater Copenhagen in Denmark. A total of 82 adults aged >50 years were screened. The first screening was conducted over the phone. Candidates were then scheduled for a home visit, during which the Montreal Cognitive Assessment test was administered by a trained neuropsychologist. The Montreal Cognitive Assessment scores were collected but are not reported in this paper as it was outside the scope of this study (see [33] for details). Of the 82 individuals, 54 (66%) (aged 52-86 years; male: 35/54, 65%; female: 19/54, 35%) fulfilled the inclusion criteria of the study. All participants signed informed consent to join the study and agreed to share their data. At a second home visit, the participants were equipped with the wearable Withings Steel HR smartwatch (Withings Inc), tracking the number of steps and heart rate on a per-minute basis. Participants were also equipped with the nearable Withings Aura sleep mattress (Withings Inc), tracking the various phases of sleep (sleep onset latency, wake, light sleep, deep sleep, rapid eye movement [REM] sleep, and waking up times) on a per-minute basis [34]. The first day of data logging for the participants spanned from June 7, 2017, to September 25, 2017. Data logging stopped for all participants on December 28, 2017. Figure 1 shows an overview of the days for which data were acquired for all participants in the study. In addition, the participants’ age, height, weight, and gender were noted upon entry into the study.

The study is based on data from the abovementioned devices—smartwatch (wearable) and sleep mattress (nearable)—and specifically the variables listed in Table 1. Most of the variables in Table 1 are either measured directly or calculated by the smartwatch during the day or sleep mattress during the night. In addition to those, we derived 2 commonly used variables in sleep research, namely, TST, which is the time in hours from falling asleep until final wake up, and sleep midtime, which is the midtime between falling asleep and final wake up.

**Table 1:** Overview and definitions of the variables used in our analysis and the self-trackers used to collect them.

Name	Description	Device
Daily step count	Number of steps during the day	Smartwatch
Diurnal heart rate–average	Mean heart rate during the day	Smartwatch
Nocturnal heart rate–average	Mean heart rate during the night	Sleep mattress
Total duration in bed	Time in hours from going to bed until getting out of bed	Sleep mattress
Total sleep time	Time in hours from falling asleep until final wake up	Sleep mattress
Sleep onset latency	Time in minutes from going to bed until falling asleep	Sleep mattress
Number of times awake	Count of how often the individual woke up during the night	Sleep mattress
Deep sleep duration	Time in hours spent in deep sleep	Sleep mattress
REM^a^ sleep duration	Time in hours spent in REM sleep	Sleep mattress
Light sleep duration	Time in hours spent in light sleep	Sleep mattress
Sleep midtime	Midtime between falling asleep and final wake up	Sleep mattress

^a^REM: rapid eye movement.

### Group-Level Analyses

To inspect long-term changes and variability at the cohort level, we considered the participants for whom data were collected the longest, starting in June 2017 until December 2017. For this part of the cohort (25/54, 46%), we calculated the daily means and SDs of the measurements.

To inspect whether there are discernable patterns in the day-to-day activities of the participants, we used *K*-means clustering that aims to group together similar numerical data, where similarity is defined through the Euclidean distance, particularly, to partition *N* observations into *K* clusters [35]. First, we applied the method using only data from the last week of the trial and then from the last 2 weeks and so on. We decided to do so because of the different starting points and to avoid seasonality effects.

**Figure 2:**
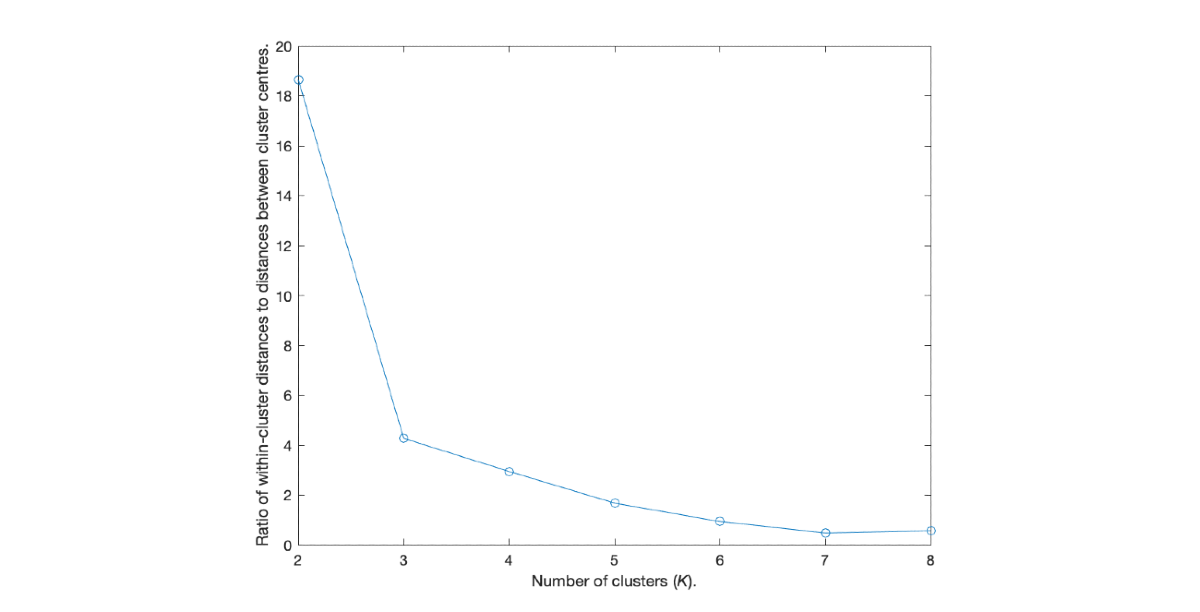
Elbow test.

To determine the number of clusters, we used the elbow method [36]. The number of clusters was chosen such that adding an additional one does not increase (much) the information gained. Specifically, we recorded the ratio of within-cluster distances of all clusters to distances between cluster centers and used Figure 2 to determine when it ceases to change (much). This created an *elbow* in the graph at *K=3*, after which not much change occurs. Note that to investigate which variables differed with statistical significance between clusters, we used the 2-sampled Student 2-tailed *t* test with *P*<.05 significance level.

### Individual-Level Analyses

**Figure 3:**
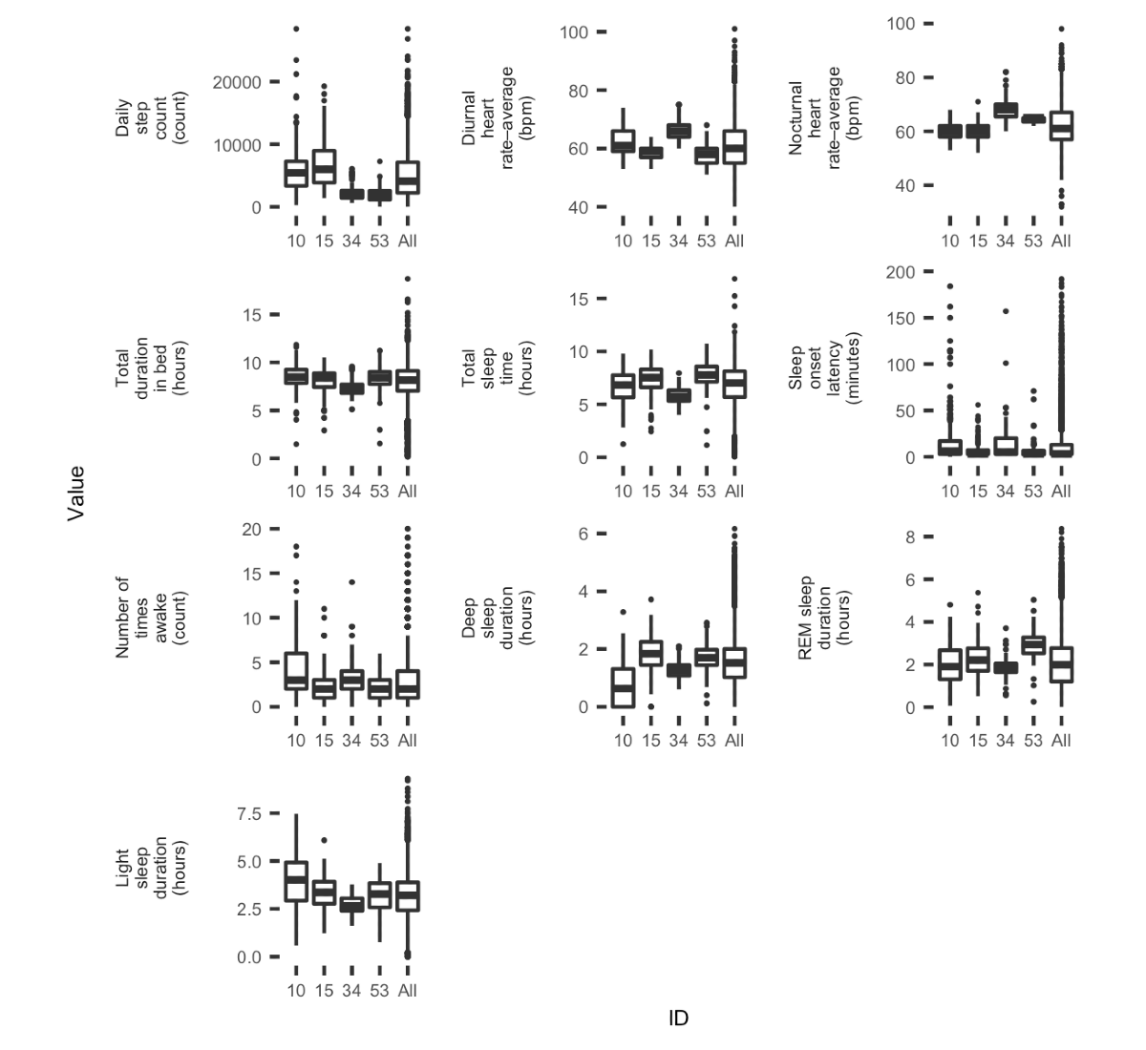
Boxplots showing the distributions for the variables considered (daily step count, diurnal heart rate–average, nocturnal heart rate–average, total duration in bed, total sleep time, sleep onset latency, number of times awake, deep sleep duration, rapid eye movement sleep duration, and light sleep duration) for the four participants and the whole population. As sleep midtime is a circular variable, it is not considered in this figure. bpm: beats per minute; REM: rapid eye movement.

To demonstrate the variability in sleep and daily activity at the individual level, 7% (4/54) of the participants were selected at random and studied in depth. Following the selection, their values were compared with those of other participants. Figure 3 shows the variable distributions of these four participants, which fell within the same range as that of the entire population. These participants were not meant to be a representative sample of the cohort, and the rationale behind our choice to show only data from 7% (4/54) of participants was to clearly demonstrate the variety in measurement patterns among participants without compromising the readability of the figures.

We considered 3 perspectives in the individual-level analyses. First, we assessed the day-to-day values for variables associated with sleep quality for a span of 1 week. The week was chosen at random. Subsequently, we calculated the weekly mean and SD for the different variables for a span of 10 weeks for the same 4 participants, where we normalized variable values for each participant by dividing by the largest value measured in the collection period. These values showed how sleep and daily activity changed from day to day and week to week.

Second, we looked at the evolution of the SD of sleep and activity measurements. We calculated a rolling SD over 7 days with a 1-day moving window from the first week of October 2017 until the end of December 2017. Moreover, starting with the first 3 days of October 2017, we calculated the SD of each participant’s measurements. Then, we added the next day and performed the calculation again. We repeated the procedure until 80 days had been added to the original 3 days. Thus, we obtained a sequence of SD values that described the variability in each participant’s measurement.

Finally, to investigate seasonal effects and, in particular, the impact of the DST clock change on October 29, 2017, we used a 2-tailed *t* test to evaluate whether differences in the values of each of the 11 variables before and after the DST clock change were significant using a .05 significance level. For this, we considered 3 periods: (1) short-term: 15 days before and 15 days after October 29, 2017; (2) midterm: 30 days before and 30 days after October 29, 2017; and (3) long-term: 60 days before and 60 days after October 29, 2017.

## Results

### Group-Level Analyses

**Figure 4:**
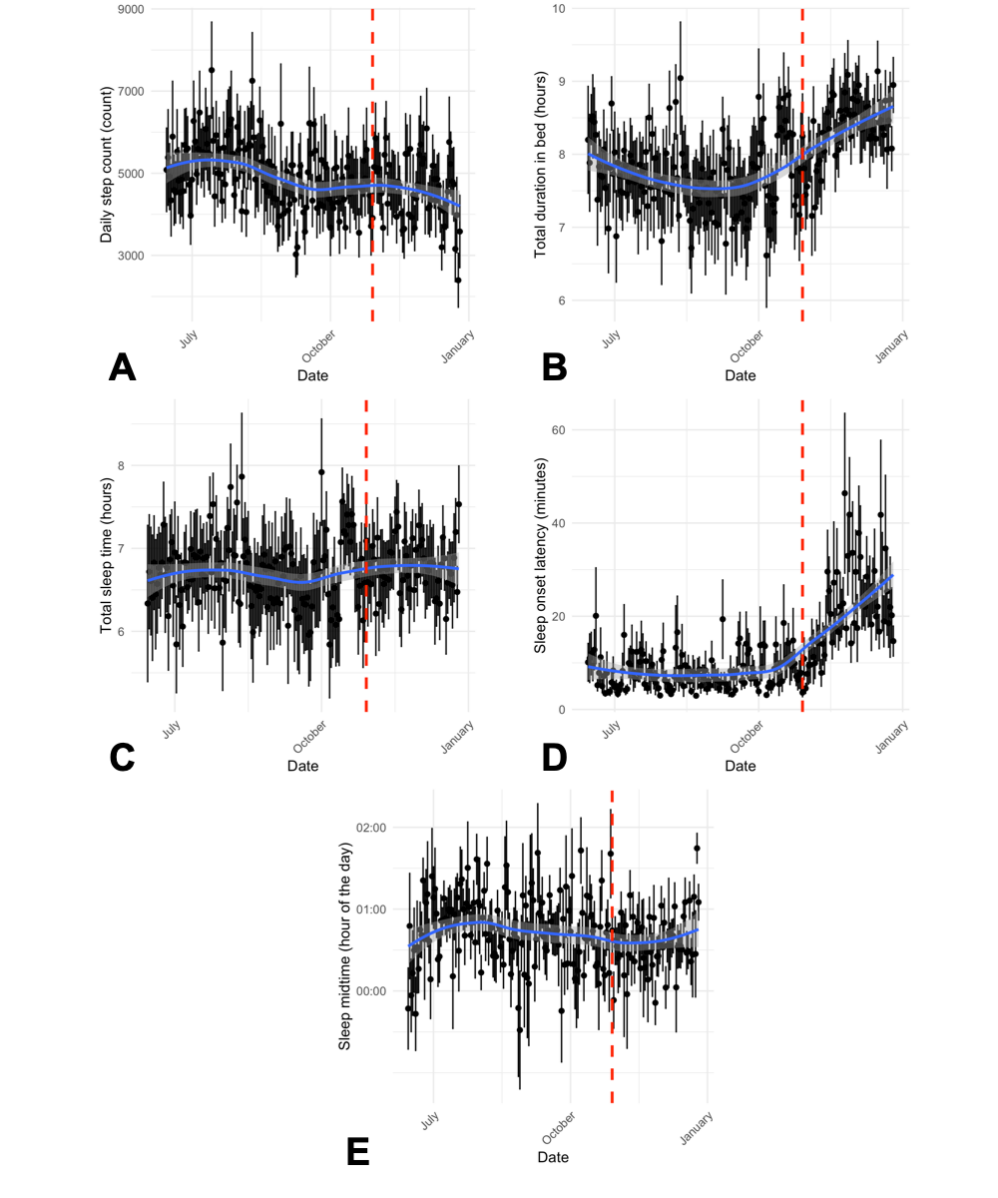
Seasonal differences in (A) daily step count, (B) total duration in bed, (C) total sleep time, (D) sleep onset latency, and (E) sleep midtime. The blue line indicates the local polynomial regression fit, and the red dashed line indicates the start of daylight saving time on October 29, 2017.

Some of the participants in the study wore self-trackers for 6 months. This allowed us to look at trends over a longer period and assess seasonal patterns. Figure 4 shows daily means and SDs for TST, total duration in bed, sleep onset latency, sleep midtime, and daily step count. These variables showed the most evidence of seasonal effects. Major trends in the data indicate that total duration in bed increased, albeit the TST remained similar. The sleep onset latency leaped at the end of October 2017, when DST stops in Europe and the clock is set back by 1 hour. Clearly, the participants in this study were affected by this change, as shown by the increased time they took to fall asleep in the weeks after the change of the clock. We also saw a downward trend in the number of steps throughout the 6-month period and fluctuations in the sleep midtime.

**Figure 5:**
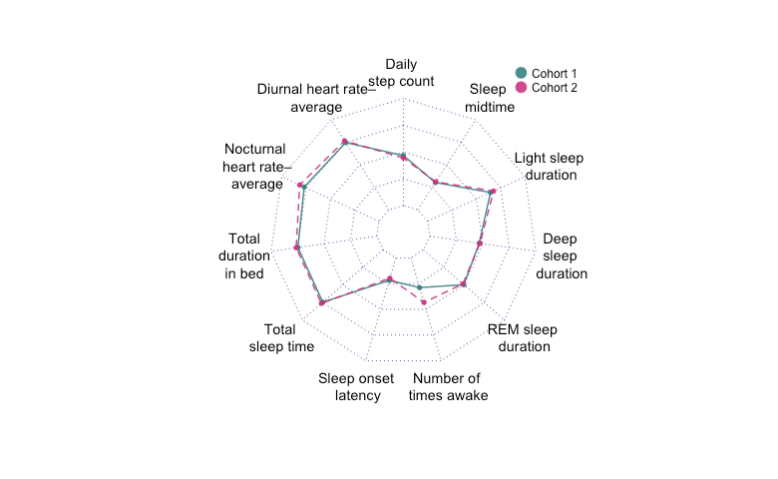
Cluster analysis revealed 2 cohorts (red and blue in the figure). The figure shows a difference in the variable number of times awake between the 2 cohorts. Other variables were less distinctive. REM: rapid eye movement.

Clustering analysis resulted in the suggestion of 2 distinct cohorts of approximately the same size (25 participants each) and a third one that we neglected for its small size when >4 weeks of data were used (Figure 5); the third cluster with 6 participants was omitted. Table 2 shows the mean and SD of all the variables in the two cohorts. The 2-tailed *t* test results showed that only differences in *number of times awake* were statistically significant. On average, the difference between the cohorts in the number of times awake was 1 time. On average, most participants woke up <4 times per night, whereas 6% (3/54) of study participants woke up >5 times per night. We did not find statistically significant differences in gender, age, or BMI between the two clusters.

**Table 2:** Mean and SD of the 11 variables in the 2 cohorts.

Variable	Cohort 1, mean (SD)	Cohort 2, mean (SD)
Daily step count (count)	4895.97 (2772.25)	4996.31 (2230.61)
Diurnal heart rate–average (bpm)	58.89 (5.91)	61.85 (6.67)
Nocturnal heart rate–average (bpm)	61.10 (6.02)	62.66 (6.85)
Total duration in bed (h)	7.81 (0.82)	8.06 (1.24)
Total sleep time (h)	6.86 (0.99)	7.08 (1.29)
Sleep onset latency (min)	10.22 (5.25)	8.70 (4.05)
Number of times awake (count)	2.03 (0.93)^a^	3.06 (1.27)^a^
Duration of REM ^b^ sleep (h)	1.54 (0.34)	1.53 (0.63)
Duration of deep sleep (h)	2.18 (0.68)	2.23 (0.77)
Duration of light sleep (h)	3.13 (0.56)	3.32 (0.68)
Sleep midtime (time)	11,249.91 (2645.61)	11,493.10 (3465.17)

^a^Indicates that the difference was statistically significant at the .05 confidence level.

^b^REM: rapid eye movement.

### Individual-Level Analyses

**Figure 6:**
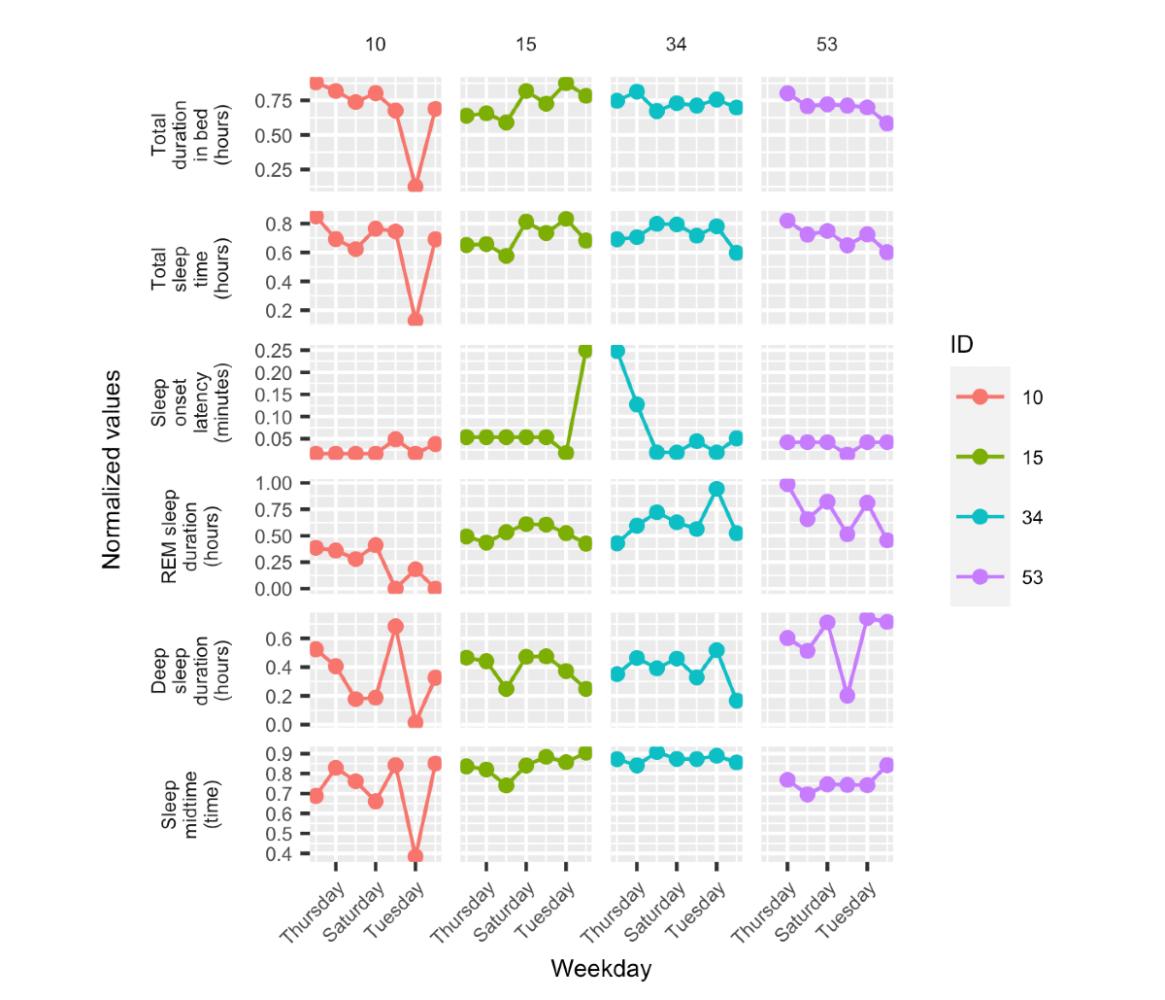
Daily parameters over a period of 1 week for the 4 participants. Each column and color represent one of the participants. REM: rapid eye movement.

Figure 6 shows time-series data for 6 variables (total duration in bed, TST, sleep onset latency, sleep midtime, deep sleep duration, and REM sleep duration) collected for 1 week for 7% (4/54) of participants on a night-to-night basis. The remaining 5 variables (daily step count, diurnal heart rate–average, nocturnal heart rate–average, number of times awake, and light sleep duration) can be seen in Figure S1 in Multimedia Appendix 1. The figures show a clear difference for each participant on a day-to-day basis and among the four of them. The relationship between the duration of REM and deep sleep differed for the participants considered here. For participant 34, they were in sync, but not for the remaining participants. Finally, sleep onset latency appeared regular for all 4 participants, and of them, 2 (50%) had days where it peaked.

**Figure 7:**
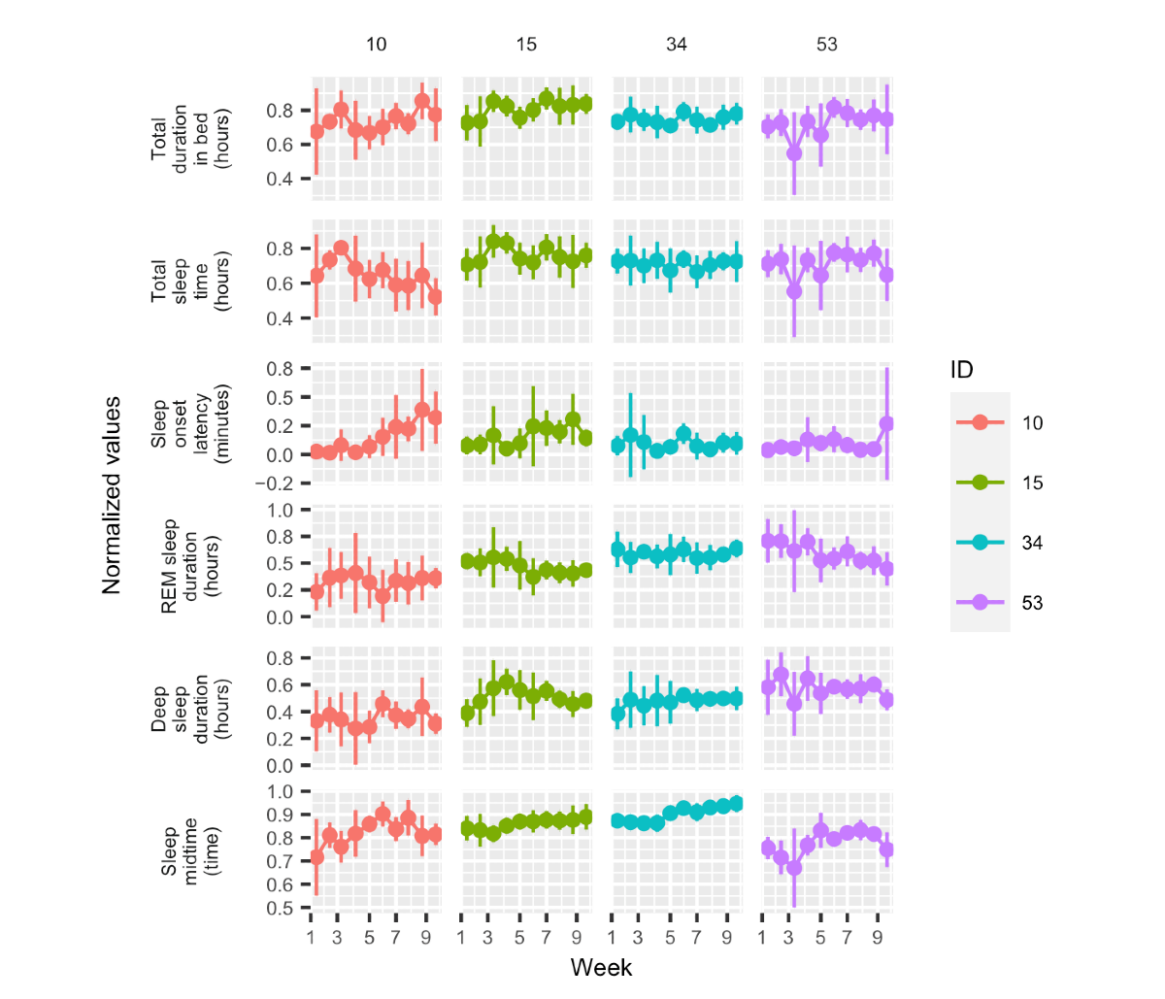
Average activity by week over a 10-week period for the 4 participants. The bars denote the SD within each week. REM: rapid eye movement.

Figure 7 shows weekly averages over a period of 10 weeks for the same 7% (4/54) of participants and 6 of the variables (total duration in bed, TST, sleep onset latency, sleep midtime, deep sleep duration, and REM sleep duration). The remaining 5 variables (daily step count, diurnal heart rate–average, nocturnal heart rate–average, number of times awake, and light sleep duration) can be seen in Figure S2 in Multimedia Appendix 1. Here, we see that the variation in measurements was even greater. For example, the sleep onset latency of participant 10 was gradually increasing, a pattern that can also be discerned in the total duration in bed and sleep midtime plots. In some weeks, the SD was large, which indicated that the values in those weeks spanned a wide range. The measurements of participant 53 showed stark fluctuation during the 10-week period.

**Figure 8:**
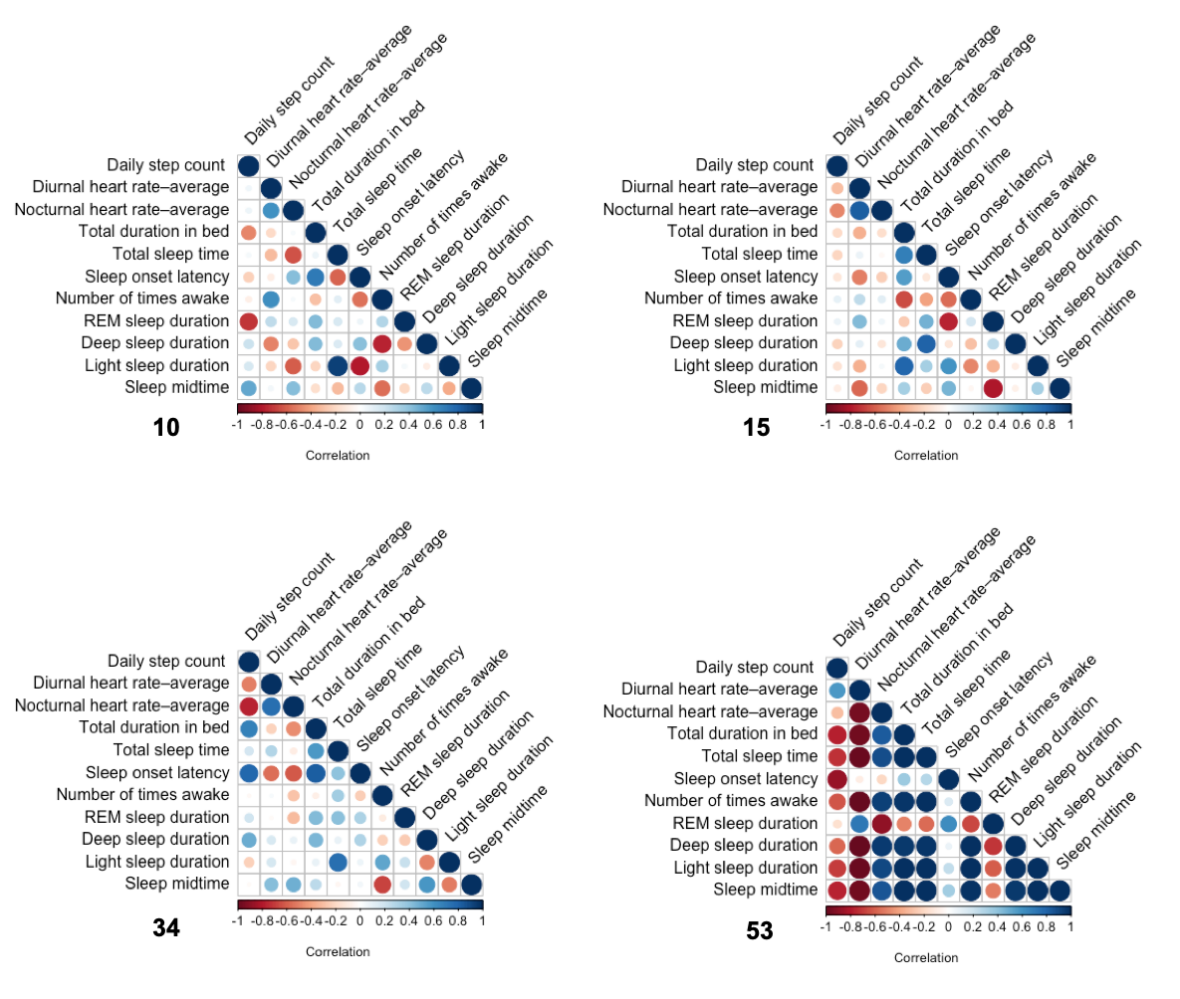
Correlation between the 11 variables for the 4 participants. REM: rapid eye movement.

Figure 8 shows the correlation between the weekly averages in Figure 7 for the 7% (4/54) of participants, which varied greatly. Participants 10, 15, and 34 had some positive and negative correlations between their variables. For example, for participant 10, there was a positive correlation between light sleep duration and TST and a negative correlation between light sleep duration and sleep onset latency. In contrast, for participant 53, most of the correlations were strongly positive or negative, showing great synergy. Only sleep onset latency showed little correlation with the other variables.

**Figure 9:**
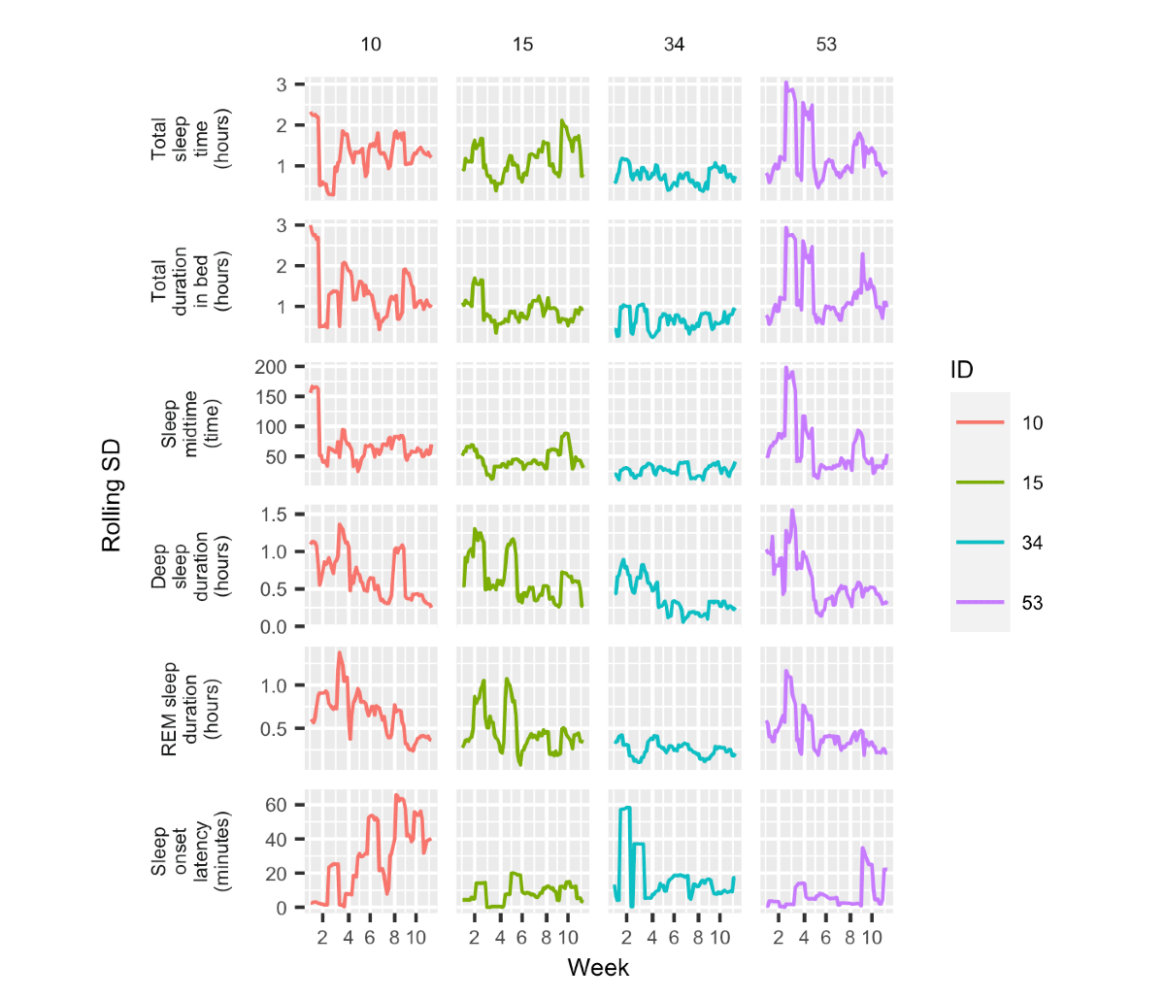
Rolling SDs over a 10-week period. These were calculated over 7 days with a 1-day rolling window. REM: rapid eye movement.

Next, we investigated the variability in different variables by focusing on the changes in SD over time. Firstly, Figure 9 shows the rolling SD computed over 1 week with a rolling window of 1 day for 6 of the variables (TST, total duration in bed, sleep midtime, deep sleep duration, REM sleep duration, and sleep onset latency). The remaining 5 variables (daily step count, diurnal heart rate–average, nocturnal heart rate–average, number of times awake, and light sleep duration) can be seen in Figure S3 in Multimedia Appendix 1. From the figure, we see that the SD changed greatly throughout time for all participants and for all measurements. Participant 34 had little variation in measurements. The SD of TST and total duration in bed remains within 1 hour. However, in the first weeks, the SD of the sleep onset latency went up to 60 minutes. The other participants had greater fluctuations throughout the period, with SD of TST reaching 3 hours for participants 10 and 53. In addition, the variability in deep and REM sleep duration decreased over time. The data also shows that the variability in sleep onset latency had an increasing trend in the 10-week period.

**Figure 10:**
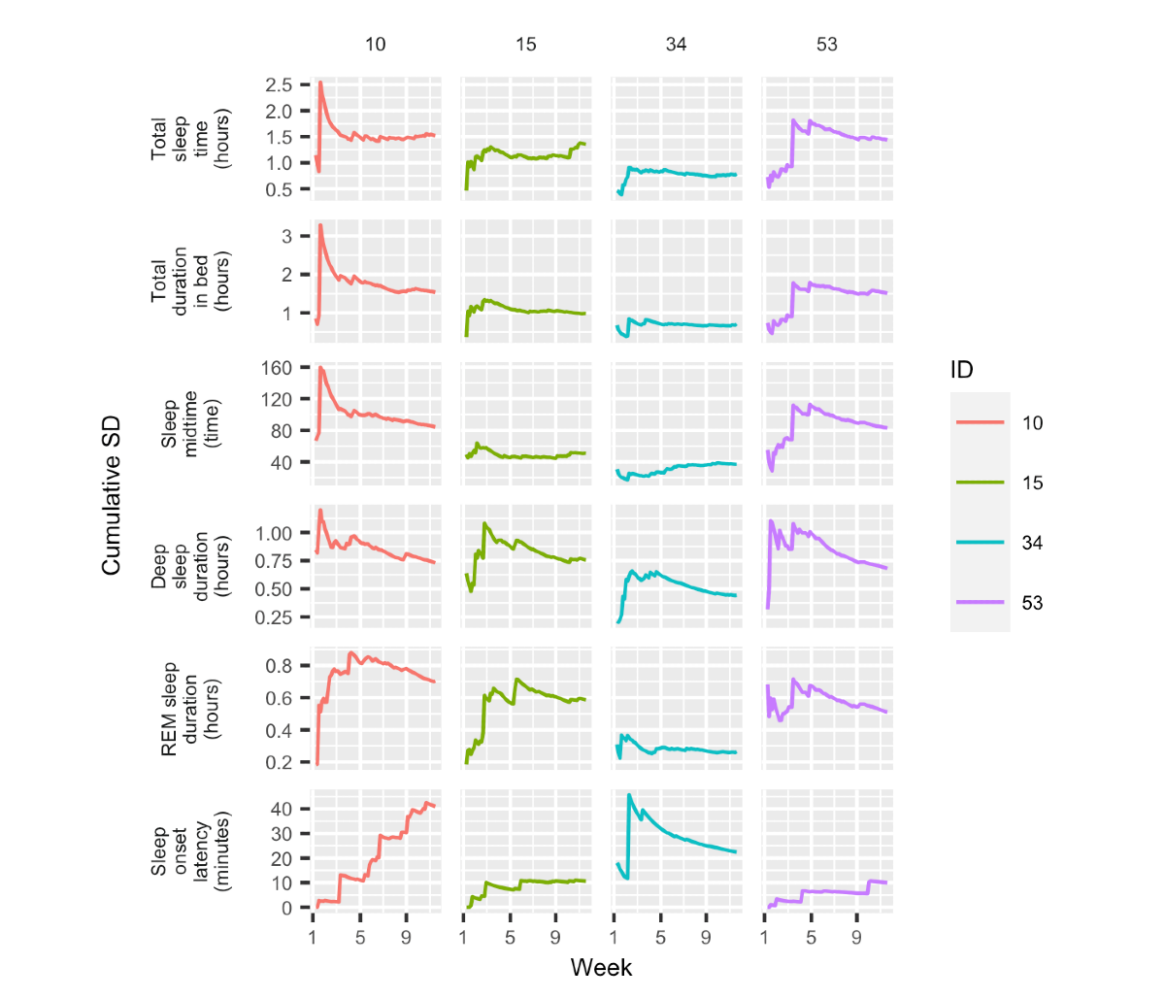
Cumulative SD over a 10-week period, adding 1 day at a time. REM: rapid eye movement.

Finally, Figure 10 shows the cumulative SD of the 7% (4/54) of participants for the same 6 variables. The remaining 5 variables can be seen in Figure S4 in Multimedia Appendix 1. These plots give a sense of the participants’ overall variability over time and how it stabilized as more days were added to the data collection. The plots show that 1 week is not representative of someone’s sleep behavior as it can change drastically from week to week.

**Figure 11:**
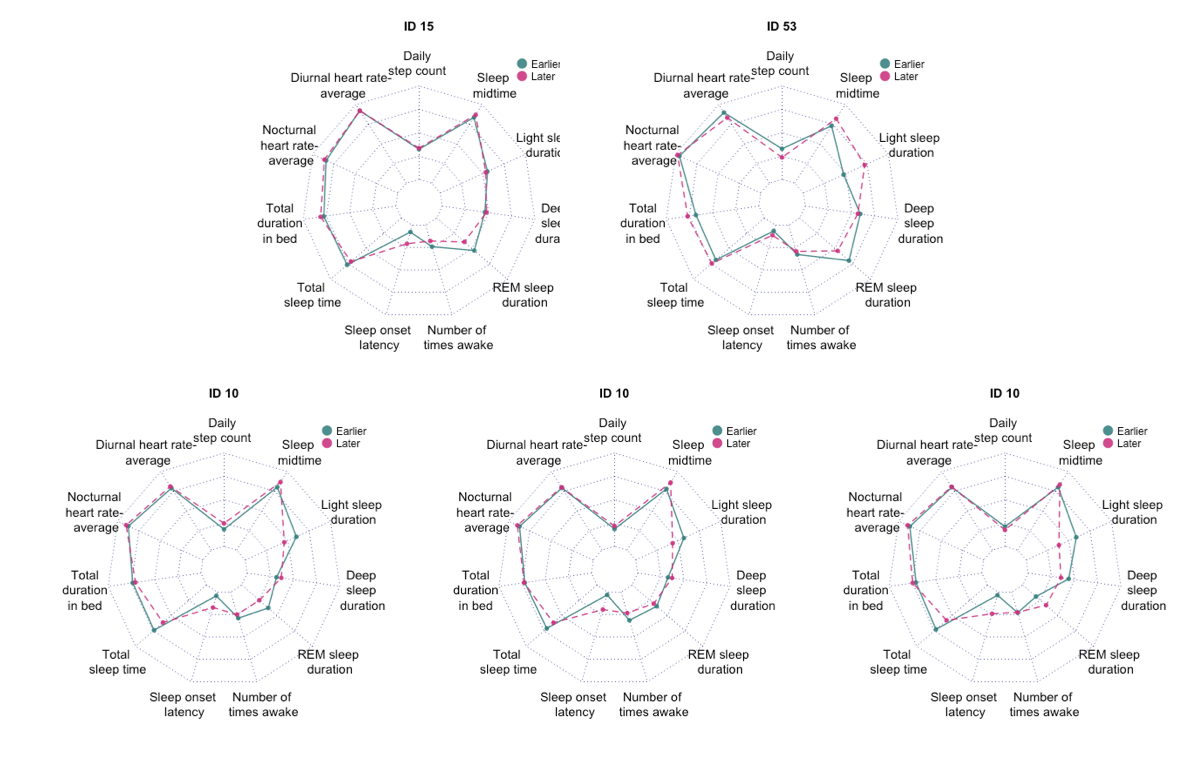
Mean values for the variables before and after October 29, 2017. Top row: long-term patterns for participants 15 and 53. Bottom row: short-term, midterm, and long-term patterns, which represent 15, 30, and 60 days before and after October 29, 2017, respectively, for participant 10. Both participants 15 (*P*<.0001) and 53 (*P*<.001) had significantly longer REM sleep before the change. Participant 10 fell asleep sooner, slept longer, and spent more time in light sleep before the change. REM sleep duration also changed from short term to long term. REM: rapid eye movement.

We now assessed the effects of seasonality on sleep and sleep quality. More precisely, we investigated which, if any, of the variables were significantly different before and after the DST clock change when looking at short-term (15 days before and after October 29, 2017), midterm (30 days before and after October 29, 2017), and long-term (60 days before and after October 29, 2017) periods for the 7% (4/54) of participants. Table 3 and Figure 11 show which variables had a significant difference before and after October 29, 2017. The difference in sleep midtime was almost always significant. Also, long-term changes were the most statistically significant, and before the changing of the clock, the participants spent more time in REM sleep, the midtime of their sleep was earlier, and they fell asleep faster.

**Table 3:** Statistical significance of the difference in variables before and after October 29, 2017, for each of the four participants during the three periods.

Variables	Short term					Midterm					Long term			
	ID 10	ID 15	ID 34	ID 53	ID 10		ID 15	ID 34	ID 53	ID 10		ID 15	ID 34	ID 53
Daily step count														✓^a^
Diurnal heart rate–average							✓		✓					✓
Nocturnal heart rate–average					✓					✓		✓		
Total duration in bed														✓
Total sleep time	✓	✓			✓					✓		✓		
Sleep onset latency	✓				✓		✓			✓		✓		
Number of times awake					✓			✓				✓	✓	
REM^b^ sleep duration							✓		✓	✓		✓		✓
Deep sleep duration												✓		
Light sleep duration	✓			✓	✓			✓	✓	✓				✓
Sleep midtime	✓	✓	✓	✓	✓		✓	✓	✓			✓	✓	✓

^a^Indicates a statistical significance at the .05 confidence level.

^b^REM: rapid eye movement.

## Discussion

### Principal Findings

Although the sleep research community welcomes the advancement of consumer-grade self-trackers, including wearables and nearables, they are also widely aware of the numerous challenges that remain, especially regarding the need to validate the devices to ensure their accuracy and reliability [8]. Although many of these barriers are for wearable and nearable technology companies to solve, it is the responsibility of the sleep research community and the medical informatics community to make a collective effort and decide upon necessary and sufficient requirements for validating the devices [31]. As we show in this paper, the duration of the validation period is an essential but grossly overlooked factor. Guillodo et al [11] acknowledged the need for long-term sleep studies, which could help identify connections between sleep quality and health outcomes. However, few attempts have been made with data from wearables and nearables. Although data collected over a longer period is essential, it is also important to make a clear distinction between group-level and individual-level approaches when it comes to research goals, clinical value, and data analysis. Although data collected over an extended period in a large cohort can reveal interesting insights about sleep patterns of the general population [10], there is much potential in using wearables and nearable devices for individualized medicine approaches as well. The approach used in this paper, where we studied individual patterns, has been fruitful for understanding sleep patterns over time.

Another shortcoming in the sleep literature is that it views and analyzes individual nights instead of analyzing time series, where trends, seasonality, and other long terms patterns can be discovered. On that basis, we showed that sleep patterns vary highly from person to person, and, because of that, an individualized approach may be more appropriate than pooling the data per night for several individuals, as is common in the literature. Moreover, we can see that the type of wearable or nearable is not the main value; instead, the main value is in comparing data from the same device, for the same individual, over an extended period. It has been acknowledged that clinical practices should embrace the unique characteristics of individual patients and their patterns and seek to individualize patient care; clearly, the same should hold for sleep [37].

In this paper, our primary research goal was to investigate whether the gold standard, the traditional time span of up to 2 weeks, is sufficient for obtaining reliable data to assess sleep duration and sleep quality of an individual when performing sleep measurements at home using wearable and nearable sensors. Our answer to this question is no. Specifically, we showed that there is much variability in the self-tracker measurements for individual participants across time. Furthermore, in our cohort analysis, we observed a clear distinction in the empirical data only when using sufficient data (>30 days) and could show the emergence of clusters that are robust to changes in the amount of data and the specific dates chosen for the analysis. However, when following individual behavior, an even longer period is needed, and we recommend >2 months.

The secondary research goal of this paper was to investigate whether there was a relationship among sleep quality, physical activity, and heart rate and whether within-group patterns in clusters of individuals exhibit similar activity and sleep patterns, both in general and in relation to seasonality. Our results show a seasonal effect on sleep patterns is related to the changing of the clock. This could both be because of overall seasonal changes and affected by the DST change, which has a significant effect on sleep patterns. This has been acknowledged previously, for instance, by international sleep and biological rhythm societies [38]. We show that there is much variability in the self-tracker measurements and apparent correlation between variables among participants [38].

### Conclusions

In conclusion, analysis and exploration of time-series data have given new insights about collecting and analyzing data from self-trackers. The findings in this paper show that it is important to get enough sleep data when attempting to understand sleep patterns from self-trackers in depth. First, the gold standard is less useful as there is much variation in the measures, both on a day-to-day basis and a week-to-week basis. This means that when collecting data on individuals, we recommend a longer period to capture as much of this variability as possible. Second, the variation in the patterns in the data is high from person to person. Although cluster analysis indicates that some patterns seem common among groups of people, our individual observations indicate that the analysis should be conducted on a person-by-person basis by training algorithms to learn individual patterns. Thus, further analysis is needed to investigate the number of days suitable for data collection with self-trackers and whether these patterns and correlations observed are common among groups of people, particularly as our analyses were only based on data from self-trackers and additional information such as illnesses, exercise plans, medication, or medical history were not included. Further limitations of the study include recruitment bias as participants were not randomly selected but were included from a homogeneous sample, and the sample size of 54 individuals affects robust conclusions. The novel finding and call to action of this paper is to reconsider the gold standard in sleep research from 14 days to >3 months. The proposition of this paper is that wearables and nearables make this possible and appear promising for clinical research under free-living sleep conditions, such as at home.

## Appendix

Multimedia Appendix 1 Additional figures showing trends in daily and weekly averages and weekly trends in cumulative and rolling SD for the five variables not included in the manuscript.

## Abbreviations

DST: daylight saving time
REM: rapid eye movement
TST: total sleep time
