# Supplementary material for: Importance of Getting Enough Sleep and Daily Activity Data to Assess Variability: Longitudinal Observational Study
Source: JMIR Form Res. 2022 Feb 22;6(2):e31807. doi: 10.2196/31807 (PMC8905485; doi:10.2196/31807)
Supplement: Multimedia Appendix 1 [file formative_v6i2e31807_app1.docx]

## Multimedia Appendix

**Importance of Getting Enough Sleep and Daily Activity Data to Assess Variability: Longitudinal Observational Study**


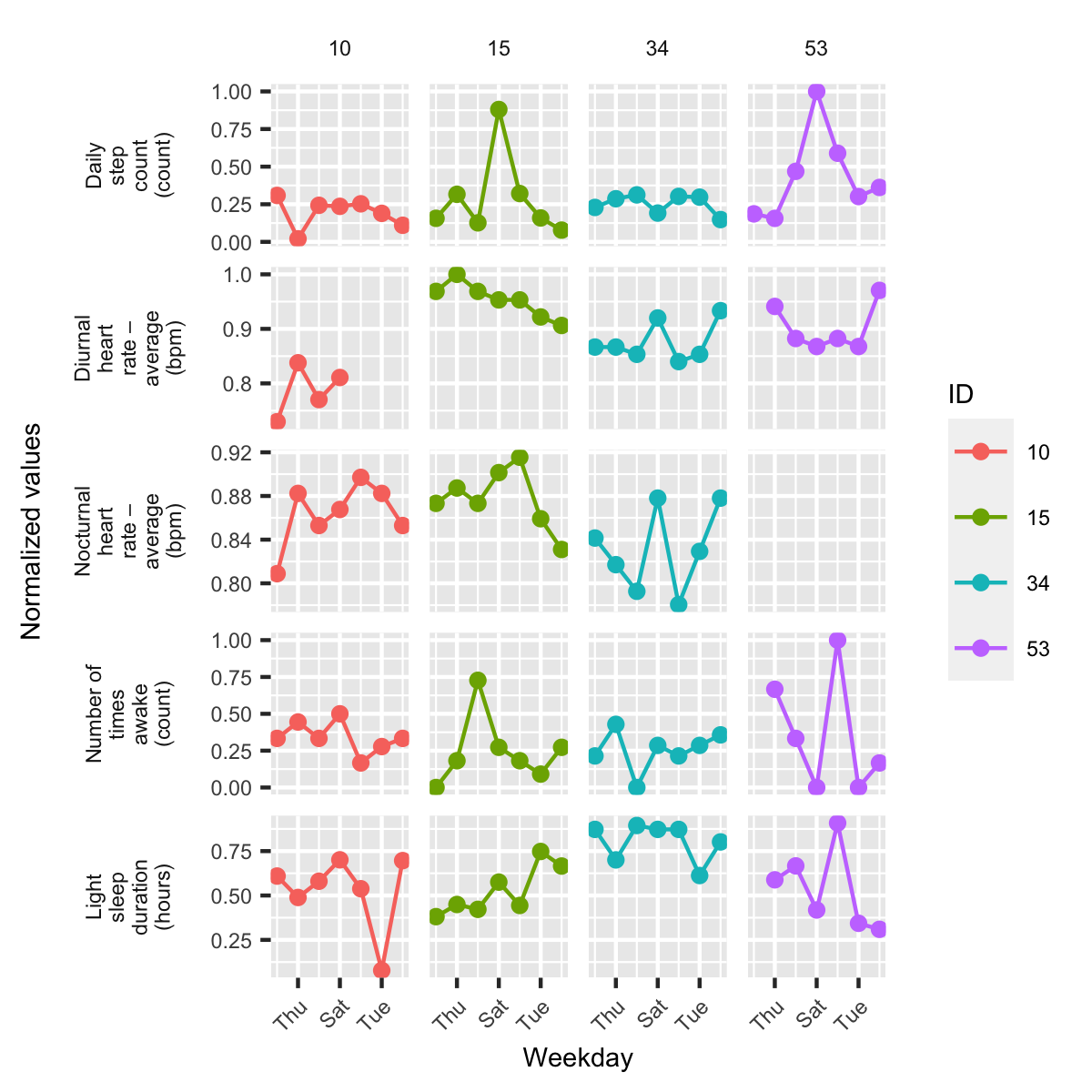
**Figure S1.** Daily parameters over a period of 1 week for the 4 participants. Each column and color represents 1 of the participants.


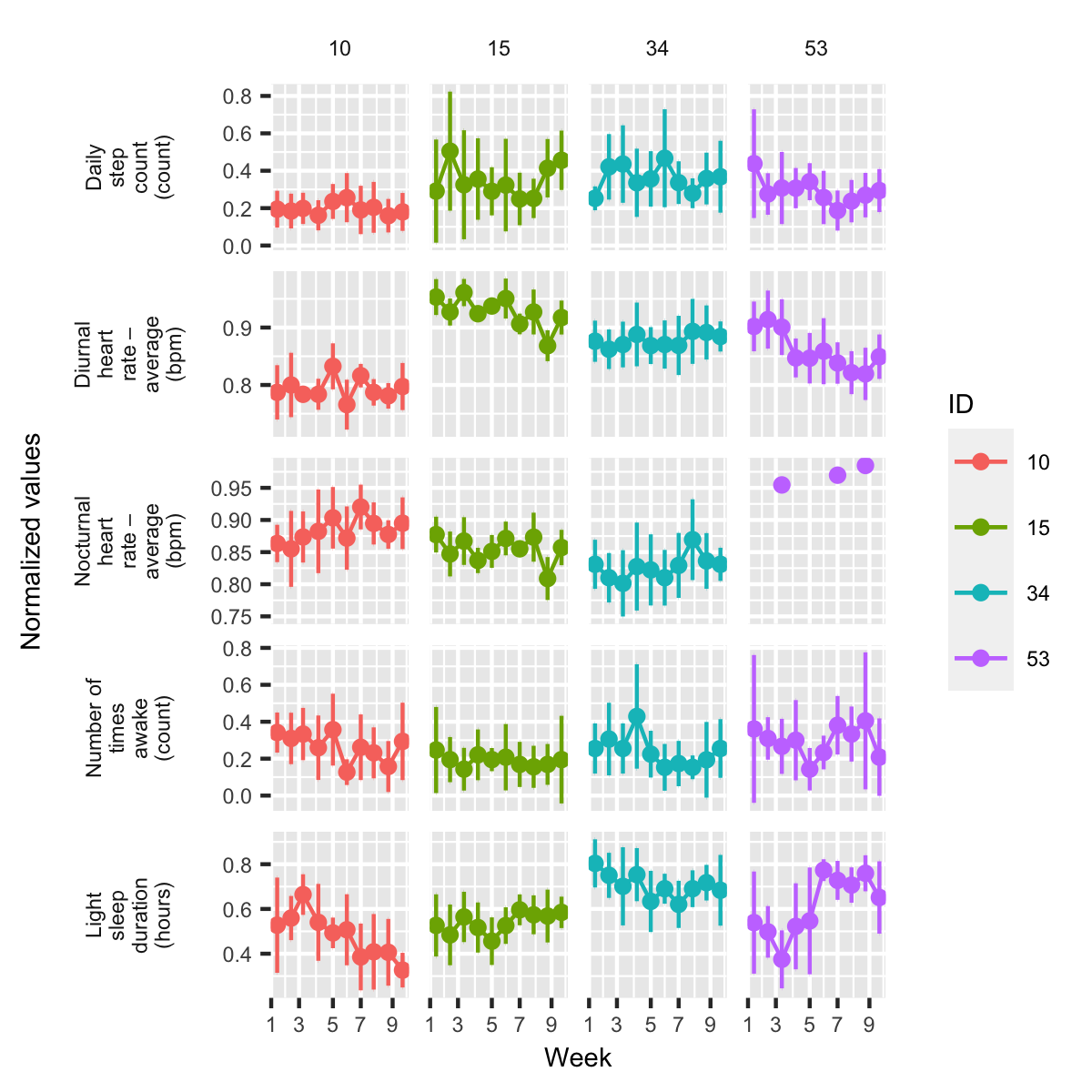


**Figure S2.** Average activity by week over a 10-week period for the 4 participants. The bars denote the SD within each week. REM: rapid eye movement.


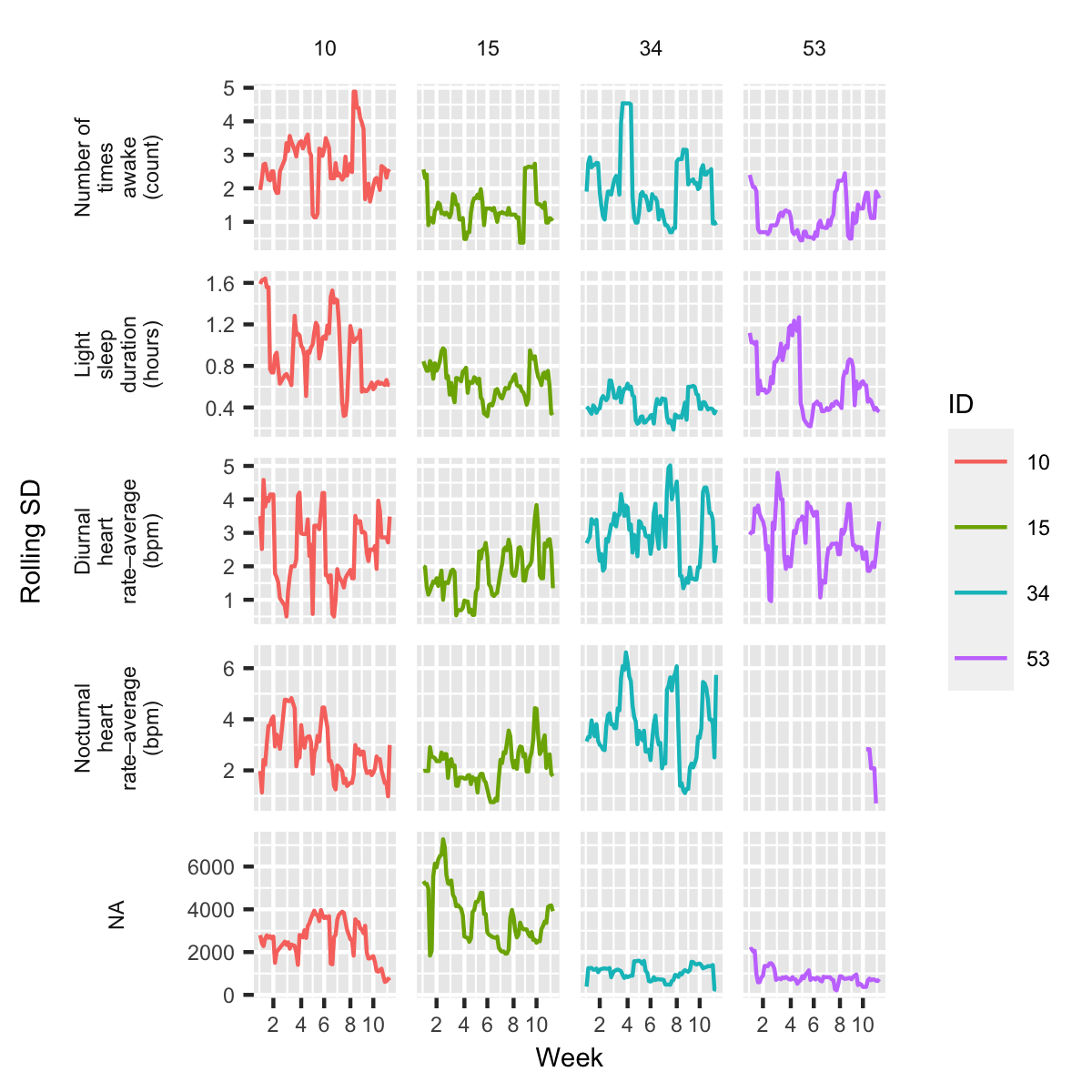


**Figure S3.** Rolling SD over a 10-week period, calculated over 7 days with a 1-day rolling window. REM: rapid eye movement.


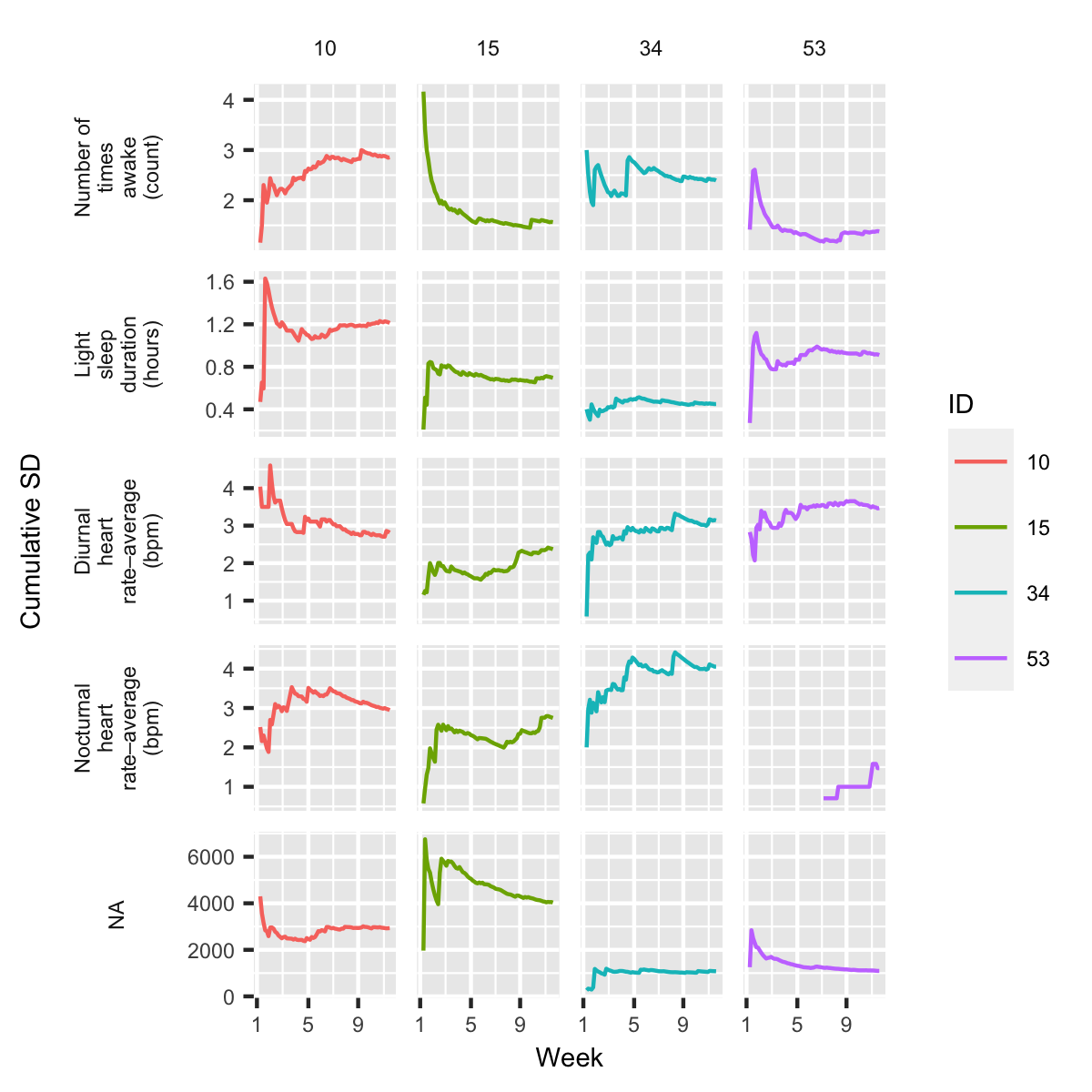


**Figure S4.** Cumulative SD over a 10-week period, adding 1 day at a time. REM: rapid eye movement.
